# Evaluation of two terpene-derived polymers as consolidants for archaeological wood

**DOI:** 10.1038/s41598-023-29785-5

**Published:** 2023-03-04

**Authors:** Michelle Cutajar, Susan Braovac, Robert A. Stockman, Steven M. Howdle, Stephen E. Harding

**Affiliations:** 1 School of Biosciences, National Centre for Macromolecular Hydrodynamics (NCMH), University of Nottingham, Sutton Bonington, Loughborough, LE12 5RD UK; 2 School of Chemistry, University of Nottingham, University Park, Nottingham, NG7 2RD UK; 3 Museum of Cultural History, University of Oslo, Kabelgata 34, 0580 Oslo, Norway

**Keywords:** Materials science, Biotechnology, Biomaterials

## Abstract

The evaluation of two terpene-derived polymers, termed TPA6 and TPA7, as possible consolidants for archaeological wood was carried out. The overall objective of this work was to expand the non-aqueous treatment toolkit which is available for the conservation of the highly degraded Oseberg collection. The wood artefacts which were found on the Oseberg ship were treated with alum in the early twentieth century, leading to the formation of sulfuric acid and to the precarious state that they are in today. Some of these artefacts cannot be treated with conventional aqueous consolidants, like polyethylene glycol, due to their highly degraded and/or reconstructed nature. This study sought to examine the level of penetration of the polymers in archaeological wood and to evaluate their consolidative effect. Both TPA6 and TPA7 were soluble in isopropanol and had a *M*_w_ of 3.9 and 4.2 kDa respectively. A number of archaeological wood specimens were immersed in solutions of these polymers. Their penetration and effects were evaluated using weight and dimensional change, colour change, infrared spectroscopy, scanning electron microscopy and hardness tests. Both polymers successfully penetrated the wood specimens, with a higher concentration found on the surface versus the core. Additionally, both polymers appeared to increase the hardness of the specimen surfaces. Increasing the polymer concentration and soaking time in future investigations could potentially facilitate the penetration to the wood cores.

## Introduction

Treating wooden artefacts with hot alum solutions was a popular method amongst conservators from the mid-1800s all the way to the 1950s^1^. This was the method that was used on the artefacts found in the Oseberg ship in the early 1900s. Unfortunately, these well-meaning conservators could not predict the damage that this treatment would eventually cause. We now know that heating alum solutions generates sulfuric acid, leading to the degraded state that these artefacts are in today^2^.

**Figure 1:**
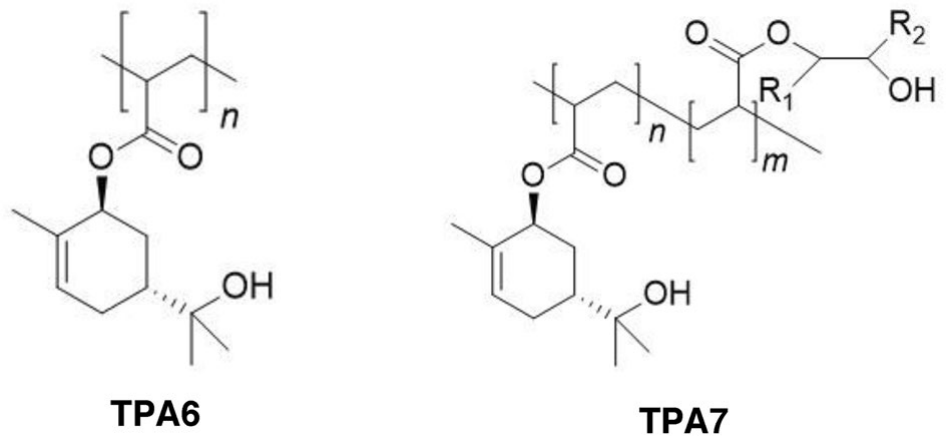
The structures for the two terpene-derived polymers that were evaluated in this study. Their synthesis and characterisation have already been described in a previous paper^13^.

In Northern Europe archaeological wood is usually found in a waterlogged state and therefore water-based conservation methods are the most effective to conserve it. The most popular conservation method usually involves treatment with polyethylene glycol (PEG) and subsequent freeze-drying^3^. If the Oseberg artefacts are retreated using this method, they would first have to be immersed in water, to remove the alum salts and acid, before adding PEG^4^. Retreating the Oseberg artefacts is not such a simple matter however, due to the large variation of the wood’s preservation state. Some of the most highly degraded artefacts cannot withstand this type of aqueous treatment as it may result in the dissolution of the alum inside the wood, leading to total collapse. Additionally, some of the artefacts contain non-wood components which were added in past restorations such as metal rods, glue and plaster^1^. For these objects, there is increased risk of damage if they are immersed in water (see Ref.^1^ and references cited therein). The Saving Oseberg project was established by the University of Oslo to safeguard these artefacts and to investigate appropriate ways with which to treat them. One of the main aims of this endeavour was to develop new treatment materials and strategies in order to have alternatives to existing consolidants (see e.g. Ref.^5^). The polymers which are commonly used in conservation nowadays are mostly derived from non-sustainable sources^6,7^. Such consolidants include Butvar® B-98, a polyvinyl butyral-based resin^6^, and Kauramin® which is a melamine urea formaldehyde resin^7^. A particular emphasis was put on the sustainability of the new materials, with the use of bioinspired polymers whenever possible. Examples of materials that have been investigated include lignin^4^, isoeugenol^8,9^, chitosan^5,10^ and aminocellulose^11^. Most recently, we have developed polymers derived from terpenes with functionalised hydroxylated moieties^12,13^. In this work we wanted to further investigate the potential of two of these polymers, TPA6 and TPA7 (Fig. 1), as wood consolidants. TPA6 is a homopolymer made from an α-pinene-derived monomer, with a *M*_w_ of 3.9 kDa. TPA7 is a copolymer derived from this same terpene monomer along with an oleic acid-based comonomer and has a *M*_w_ of 4.2 kDa. The *T*_*g*_ values for TPA6 and TPA7 were measured to be 31.1 °C and 18.9 °C respectively^13^. Both are yellow solids at room temperature and 50 g of each polymer was available for the wood testing. Since the two polymers have low *M*_w_ values, it was envisaged that they would be able to successfully penetrate wood. Moreover, they both possess hydroxyl groups which were anticipated to aid in the hydrogen bonding taking place between the consolidants and the wood. The main aim of this investigation was to determine the extent to which these polymers penetrate wood after immersion in isopropanol and to determine if they provided any consolidative effect. Isopropanol was deemed a good solvent to use in such studies since it has an acceptable safety profile and had been previously used in conservation experiments with degraded alum-treated wood^14^. Moreover, volatile solvents like isopropanol are particularly useful for wood impregnation since they can efficiently transport the consolidant polymers and then evaporate rapidly after treatment^15^.

## Materials and methods

### Preparation of wood samples

Cube specimens were made from waterlogged archaeological wood which was the property of the University of Oslo's Museum of Cultural History. The wood, identified as *Pinus* (pine) with light microscopy, was initially in the form of a branch. The branch (diameter ~ 8 cm) did not contain heartwood, which would have been darker than the surrounding sapwood. The pith was centred in the branch, which indicated there was likely no reaction wood. Furthermore, the branch was evenly degraded, evidenced by a lack of difference in resistance of surface and core when pierced with a pin during preliminary examinations. Even degradation was also reflected in density measurements (as discussed below), which did not indicate differences between surface and core samples.

The branch was sawn into six slices, with each slice then being cut into 2 × 2 × 2 cm^3^ cubes, where sides were aligned with radial and tangential directions (Fig. S1). These were freeze-dried and then acclimatised to 20 °C and 50% RH. A total of 30 cubes of archaeological wood were used for these experiments: 20 for immersion tests with the polymer solutions and the remaining 10 for the two control groups. In addition, the leftover pieces of wood that remained after the cutting of the cubes were used for density and maximum water content measurements.

### Naming system

The wood specimens were named in a way so that one could identify which slice they were cut from, and whether they were a surface or a core sample. Generally, the ID for each specimen was comprised of three characters: the first indicates their slice number (1–6), the second denotes whether they were cut from the surface or core (S = surface; C = core) and the last is a nominal specimen number. For each slice, eight cubes originated from the surface and two cubes from the core (Fig. S1, right).

### Density and maximum water content measurements

For these studies, 6 wedges from each of the six wood slices were used: 4 sawn from the surface from each slice and 2 from the core. This was done to be able see whether the wood slices were degraded to different degrees and to observe whether there was any difference between the surface and the core of the wood. The basic density was determined by dividing the oven-dried weight by waterlogged volume (Eq. 1), following the notation of Jensen and Gregory^16^:

1 $$\rho_{{{\text{so}}}} = \, \left( {M_{{{\text{ss}}}} /M_{{{\text{up}}}} - { 1}} \right)/\left( {{1}/\rho_{{{\text{lpor}}}} - { 1}/\rho_{{{\text{ms}}}} + R_{{{\text{sorp}}}} /\rho_{{{\text{lpor}}}} } \right),$$

where *M*_ss_ = mass of waterlogged sample (g); *M*_up_ = mass of displaced volume of waterlogged sample (g); *ρ*_lpor_ = mass of water in pores per volume of pore water at 20.0 °C (0.99823 g/cm^3^); *ρ*_ms_ = mass of cell wall material per volume of cell wall material (g/cm^3^); *R*_sorp_ = correction factor due to sorbed water (0.028).

The waterlogged volume was determined from the weightmass) of water displaced by the waterlogged samples. The *ρ*_lpor_ was assumed to be equal to the density of free water at 20.0 °C (0.99823 g/cm^3^)^16^. The *ρ*_ms_ value used in Eq. (1) was calculated using Eq. (2), again following the notation of Jensen and Gregory^16^:

2 $$\rho_{{{\text{ms}}}} = \rho_{{{\text{lpor}}}} /\left\{ {{1 } + \, \left( {W_{{{\text{up}}}} - W_{{{\text{ss}}}} } \right)/\left( {M_{{{\text{ms}}}} + R_{{{\text{sorp}}}} } \right)} \right\},$$

where *ρ*_lpor_ = mass of water in pores per volume of pore water at 20 °C (0.99823 g/cm^3^); *W*_up_ = weight of displaced volume of waterlogged sample and surface water (g); *W*_ss_ = weight of waterlogged sample and surface water (g);* M*_ms_ = mass of cell wall material (g); *R*_sorp_ = correction factor due to sorbed water (0.028).

The maximum water content (MWC) percentage was also determined at the same time and is expressed as the weight of water contained in the sample relative to the oven-dried weight (Eq. 3)^16^:

3 $${\text{MWC}}_{{}} = \, \left\{ {\left( {M_{{{\text{swet}}}} - M_{{{\text{ms}}}} } \right)/M_{{{\text{ms}}}} } \right\} \, \times { 1}00,$$

where MWC (%) = mass of maximum water per mass of cell wall material (g/g); *M*_swet_ = mass of wood in saturated condition; *M*_ms_ = mass of wood in anhydrous condition, obtained by placing the sample at 103 °C in an oven for at least 24 h.

### Immersion tests with polymer solutions

The wood cubes were divided into two control groups and two treatment groups (Table 1). The cubes in control group 1 were freeze-dried only and kept at 50% RH throughout the entire study. This control group was used to estimate the error range of the measurement method that was used to determine linear dimensional change (radial and tangential). Those in control group 2 were freeze-dried and then immersed in isopropanol. The specimens in treatment group 1 and 2 were freeze-dried and then immersed in polymer solutions of TPA6 and TPA7 respectively. The specimens were laid on their sides, to promote infusion from the transverse faces during immersion, which are the most easily penetrated. The beakers were then sealed to avoid evaporation using a watch glass and Parafilm. The solution level was monitored by tracing the surface of the impregnation solution after samples were saturated with it (a few hours) on the beaker; the solution level did not change over the 2-week impregnation period.

**Table 1:** The IDs of the wood specimens in each group used in this study.

Control group 1—freeze-dried only	Control group 2—isopropanol	Treatment group 1—TPA6	Treatment group 2—TPA7
1.S.6	2.S.7	4.S.3	4.S.6
2.S.4	3.S.5	1.S.4	1.C.2
4.C.1	4.C.2	2.S.1	2.S.2
5.S.5	5.S.3	2.C.2	2.S.3
	6.S.6	3.S.1	3.S.2
		3.S.4	3.S.7
		4.S.2	4.S.5
		4.S.4	4.S.1
		5.S.1	5.S.2
		6.S.1	6.S.4

The two polymers were dissolved in isopropanol at a 15% w/w concentration. This concentration was decided upon as a result of preliminary tests which had been carried out in order to determine the best testing concentration. This involved using mixtures of wood powder and different concentrations of polymer/isopropanol solutions to make thin films of wood/polymer. Concentrations of 5–15% were tested and the highest concentration appeared to impart the most strength, meaning that the concentration of 15% produced the most robust films. Because of this, it was decided to use this concentration in these immersion tests. Higher concentrations were not tested due to the limited amount of polymer that was available. Both polymers appeared to dissolve completely in this concentration, therefore full dissolution was assumed. These solutions were then used to soak the assigned wood specimens. Control group 2 as well as both treatment groups, were left immersed in their respective solutions for 2 weeks, based on a previous report of wood testing with non-aqueous treatments^4^. The wood cubes were then removed from the solutions and left to slowly air-dry in a fume-hood for 1 week, after which they were kept in climate chamber at 20 °C and 50% RH.

### Change in weight and dimensions

Weight (mass) was measured using a four decimal balance. The weight of the cubes was reported before (after freeze-drying and after conditioning to 20 °C and 50% RH) and after treatment (after evaporation of solvent and after conditioning to 20 °C and 50% RH).

Dimensions were measured with digital callipers, using pins inserted into the cross-section of the cubes along radial and tangential directions. Changes in the longitudinal direction are minor in comparison. Both the radial and the tangential directions, referred to as ‘linear dimension’, of control group 1 (freeze-dried only) and of the treated specimens were measured before and after treatment. All measurements were taken after freeze-drying and acclimatisation to 20.0 °C and 50% RH, such that it was possible to compare change from the same point of reference. The reason for measuring radial and tangential directions of specimens in control group 1 was to estimate the error involved in this measurement technique, since in theory there should not be differences in the radial and tangential dimensions when stored under constant climatic conditions. The % weight change and % linear dimensional change were calculated for the treated groups using Eqs. (4) and (5):

4 $$\mathrm{\% Weight} \, \mathrm{change }=\frac{(\mathrm{weight} \, \mathrm{after}-\mathrm{weight} \, \mathrm{before})}{\mathrm{weight} \, \mathrm{before}} \times 100\%,$$

5 $$\mathrm{\% Linear} \, \mathrm{dimensional} \, \mathrm{change }= \frac{(\mathrm{dimension} \, \mathrm{after}-\mathrm{dimension} \, \mathrm{before})}{\mathrm{dimension} \, \mathrm{before}} \times 100\mathrm{\%}.$$

### Colour change

Colour measurements were taken of all treatment groups and control groups. This was carried out with a Konica Minolta CM-700d handheld spectrophotometer, making use of the CIELAB (L*a*b*) colour space. The L* coordinate measures the lightness/darkness of the samples, on a scale from black (−) to white (+). The a* coordinate indicates a colour change from green (−) to red (+) and the b* measures a colour change from blue (−) to yellow (+). The light source used was D65 (daylight), the measurement diameter was 4 mm and the data provided was Specular Component Excluded (SCE). Colour measurements were taken of the longitudinal sides, two random spots were measured on each side. However the specimens’ dark sides—if present—were not included. This amounted to six to eight measurements per specimen. Measurements from each control and treatment group were then averaged for each coordinate (L*, a*, b*). The change in colour caused by TPA6 and TPA7 was calculated from the difference of the averaged group measurements (ΔL*, Δa*, Δb*) between each of the two treatment groups and control group 2 (wood treated with just isopropanol). The absolute change, ΔE*, was finally calculated from the coordinate measurements using Eq. (6):

6 $$\Delta {\mathrm{E}}^{*} = {(\Delta {\mathrm{L}}^{*2} +\Delta {\mathrm{a}}^{*2} +\Delta {\mathrm{b}}^{*2})}^\frac{1}{2}.$$

### Attenuated total reflection-Fourier transform infrared spectroscopy (ATR-FTIR)

Fourier transform infrared spectroscopy was carried out using an attenuated total reflection mode on a Thermo Fischer FTIR spectrometer (Nicolet iS50), with range 4000–400 cm^−1^. The samples used for this part of the study included those from sound pine, an isopropanol-treated control, the treatment groups, as well as pure polymer samples. A resolution of 4 cm^−1^ was used and each spectrum was derived from 32 scans. Three spectra from each sampling site were taken and averaged. The wood samples were analysed at four points: the surface, the core and between the surface and core both along and across the grain (Fig. S2). Thermo Scientific OMNIC FTIR software was used to analyse the data. Spectra were baseline corrected and normalised to 1508 cm^−1^ (wood spectra) or 1725 cm^−1^ (pure polymer spectra).

### Scanning electron microscope (SEM) analyses

A FEI Quanta 450 Scanning Electron Microscope with a voltage of 8 kV on low vacuum was used. Spot size was 4.5, chamber pressure was 50–80 Pa with working distances of 7.0–10.0 mm. The SEM samples were carefully shaved with a razor in order to get a surface which was flat, enabling better visualisation of the wood cells. It was ensured that not too much material was shaved off from the surface, in order not to compromise the results. No coating was required since a high vacuum was not used.

### Resistance to indentation (hardness test)

A fruit penetrometer (Lutron FR-5120) motorised with a TRINAMIC Motion control engine (Model TMCS-40.6.35-10000-AT-01) with 10,000 P/R resolution was utilised (Fig. S3). The penetrometer was fitted with a 3 mm radius tip which was used to apply a force on the tangential face of the wood specimens. The motor was operated through a hardwired controller. It was ensured that the wood specimens were lying on a flat surface and that the penetrometer’s tip touched the wood’s testing face perpendicularly. The testing faces of the wood specimens were shaved with a razor in order to ensure that they were flat and parallel. This was not always possible however, as a balance had to be struck between how much material was shaved off and the flatness of the testing faces. A specimen of sound pine was first measured. The wood specimens in the control groups were all measured. Eight specimens were measured from each treatment group. Each wood specimen was measured five times on its tangential surface and the values were then averaged (Fig. S3). Additionally, each wood specimen from the treatment groups were split in two and the core was measured once on each half and averaged (Fig. S3). Prior to each measurement, a preload was applied by lowering the penetrometer tip on to the testing face of the wood specimen until the force read by the sensor was between 6 and 10 N. For the actual measurement, the tip was lowered down 1 mm and the force in N was recorded. The net force was calculated by subtracting the preload force from the measured force. IBM® SPSS® software was used to analyse the data with a one-way ANOVA test to measure whether the differences in hardness were significant.

### Ethical approval

All procedures were conducted in accordance with the relevant guidelines. Archaeological *Pinus* wood was supplied by the Museum of Cultural History in Oslo and had been stored in a waterlogged state until use. Collection and use of this wood did not require permission.

## Results and discussion

### Density and maximum water content (MWC) measurements

The density and MWC of wood are both important indicators of its degradation. Knowing the density of the wood can give us an idea of the amount of consolidant required to conserve it^16^. The water content of waterlogged wood is referred to as MWC since it is assumed that wood which has been preserved in such conditions would possess the highest possible volume of water in its pores^17^. The MWC increases and the basic density decreases with increasing wood degradation, which results from the loss of mass and volume of cell wall material due to bacterial degradation taking place during burial^18^.

From the density and MWC measurements, it appeared that the state of preservation of our wood branch was approximately even throughout, that is, there was no difference between surface and core specimens. The density measurements ranged from 0.132 to 0.179 g/cm^3^. Sound pine is reported to have a density of around 0.5 g/cm^3^^19^. The MWC ranged from 492 to 653% indicating a high degree of degradation for pine, since above 250% water content it is considered to be degraded^20^. Based on this, our wood samples seemed to have a high degree of degradation, with the means of the density and MWC being 0.146 g/ cm^3^ and 610% respectively. Moreover, the samples taken from the surface and the core of our wood branch appeared to show little difference in both density and MWC, meaning that they had similar preservation states.

### Macroscopic observations

After treatment, the specimens treated with both polymers seemed darker, with a yellowish tinge. They felt denser when handled. There did not appear to be any new cracks that developed on the wood after treatment and they kept their original shape. This was ascertained by looking at photos taken before and after treatment (see Fig. 3a,b as examples).

### Weight change

**Figure 2:**
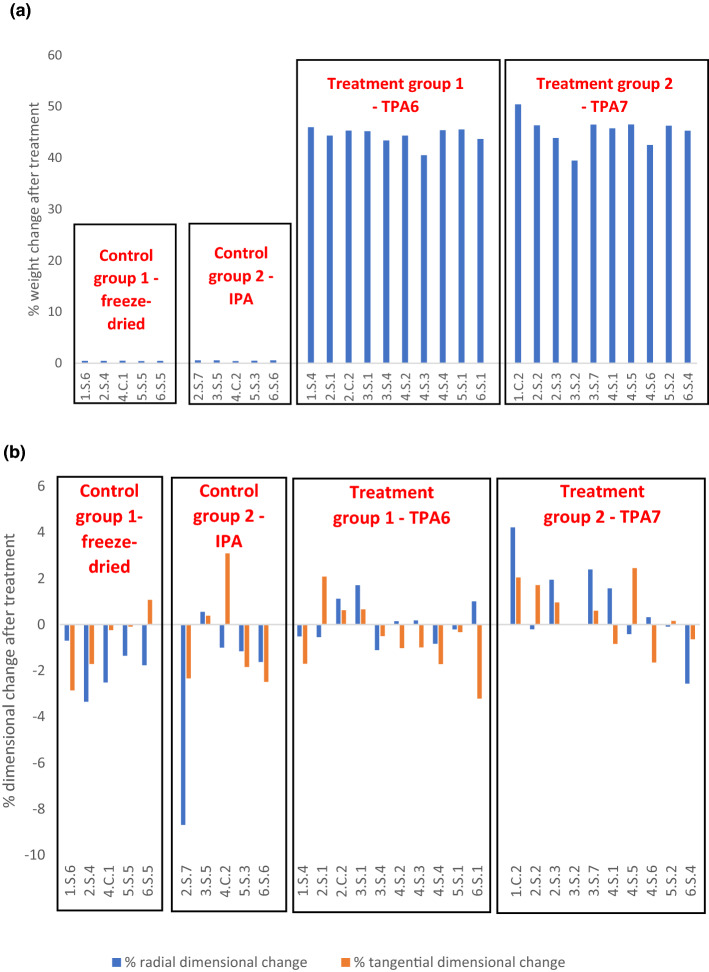
(**a**) The % weight change for all the measured groups. Control group 1 was freeze-dried and kept in a desiccator at ~ 20 °C and 50% RH during the immersion of control group 2 (isopropanol-immersed) and treatment groups 1 and 2 (TPA6- and TPA7-immersed respectively). (**b**) A comparison of the radial and tangential dimensional % changes for control groups 1 and 2 and treatment groups 1 and 2.

The specimen groups treated with TPA6 and TPA7 were observed to substantially increase in weight (Fig. 2a). The control group treated with isopropanol (referred to as IPA in Fig. 2a) was expected to decrease in weight due to the dissolution of some pine resin in the solvent. During immersion, the solvent was observed to turn from transparent to a pale yellow, possibly indicating that some resin may have exited the wood. This was not found to be the case however since the specimens actually increased in weight, albeit very slightly. It is possible that these cubes were not conditioned to the same extent before and after isopropanol immersion, thus leading to a slight irregularity in the weight measurements.

Weight gain appeared to be uniform for the specimens treated with TPA6 and TPA7. TPA6 had a % weight change ranging from 40.5 to 45.9% while TPA7 ranged from 39.5 to 50.4%. The specimen with the most weight gain overall (50.4%) was a piece from the core treated with TPA7 (1.C.2). The sample showing the least weight gain (39.5%) was also from the TPA7 group (3.S.2). It is unclear why the polymer deviated in its penetration for these two particular specimens. Since the wood specimens were conditioned both before and after treatment, then this meant that these differences in penetration were not influenced by temperature or the relative humidity. Taking this into consideration, TPA6’s penetration into the wood appeared to be slightly more regular with no extremes. It is uncertain whether this is due to the polymer’s properties, or if it is an effect of the wood’s inherent variability.

### Dimensional change

For all groups, the dimensional change for both radial and tangential faces primarily ranged at ± 2%, except for a few outliers (Fig. 2b). Both radial and tangential faces seemed to change to the same degree, that is, one face did not predominate over the other. Control group 2 appeared to shrink after isopropanol treatment. The treatment groups had more varied results, showing both shrinkage (negative values) and swelling (positive values). TPA6 seemed to mainly cause shrinkage. TPA7 seemed to have a more variable effect, with swelling slightly predominating over shrinkage. As mentioned, there were a few outliers in these measurements. The most extreme was 2.S.7 (control group 2), which showed a radial dimensional change of − 8.7%. Other outliers include 4.C.2 (control group 2; tangential change of 3.1%), 6.S.1 (treatment group 1; tangential change of − 3.2%) and 1.C.2 (treatment group 2; radial change of 4.2%). These measurements may potentially be attributed to the high variability of the wood or they may also be due to experimental error. The ‘pin method’ of measuring dimensional changes has limitations when it comes to the accuracy of the measurements. This is because the measurement values are highly dependent on the angle of the calliper relative to the pins. Therefore, if this angle is not exactly replicated during the ‘before’ and ‘after’ measurements, it will be difficult to get results that truly represent the dimensional change that has taken place.

With regards to control group 1 (freeze-dried only), the specimens were kept in a desiccator at ~ 20.0 °C and 50% RH during the soaking of the other groups. Its measurements were then repeated after the treatment of the other specimens was completed and after they were acclimatised to 20.0 °C and 50% RH. This means that theoretically the specimens in control group 1 should have exhibited no dimensional changes since they were not subjected to any treatment after freeze-drying. Nevertheless, slight dimensional changes were still recorded (~ ± 2%). This demonstrates the inherent inaccuracy of this measuring method. The dimensional changes recorded in control group 2 may therefore be considered as the error range that can be expected for the other measurements (~ ± 2%). If we take this into consideration, then this means that most of wood specimens did not exhibit significant dimensional changes after treatment.

### Colour change

**Figure 3:**
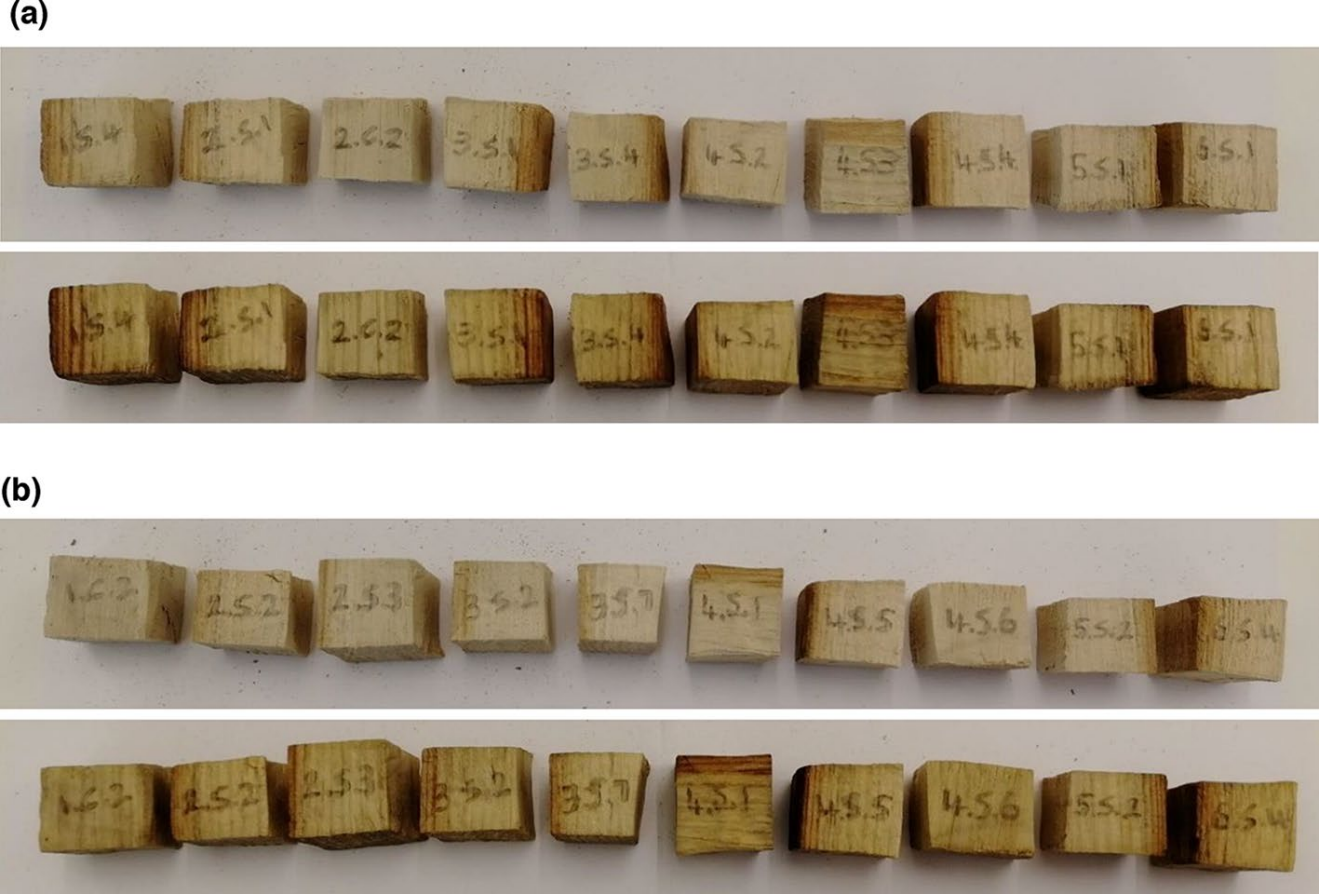
(**a**) Photographs of treatment group 1 (TPA6) before (top) and after (bottom) treatment. Specimens became noticeably darker, with a yellow tinge. (**b**) Photographs of treatment group 2 (TPA7) before (top) and after (bottom) treatment. Specimens were similar to those treated with TPA6.

**Figure 4:**
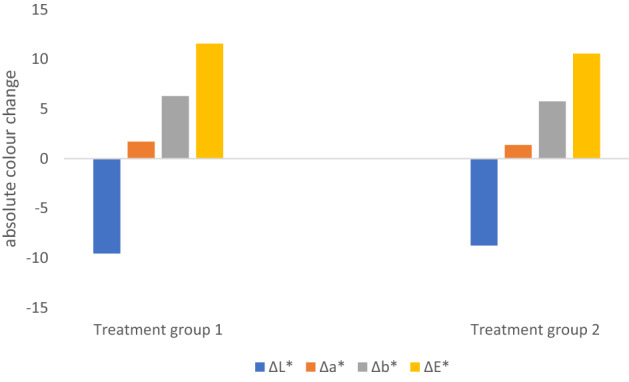
Colour change of treatment group 1 (TPA6) and treatment group 2 (TPA7) relative to control group 2 (isopropanol-immersed). Treatment group 1 ΔE = 11.55; treatment group 2 ΔE = 10.55.

Based on visual inspection alone, it was obvious that the polymers had caused a colour change in the wood specimens, mostly darkening them and imparting a yellow tinge (Fig. 3). This is possibly a result of the polymers imparting their colour to the wood. The spectrophotometer colour measurements of both treatment groups were compared to those of control group 2, which was treated with isopropanol (Fig. 4). Both polymers seemed to change the colour to a similar degree, as was observed visually. The measurements show that TPA7 seemed to cause slightly less colour change than TPA6, although this could not be seen by the naked eye. The largest degree of change was observed in the L* coordinate which decreased after the treatments, meaning that the specimens became darker. There was also a substantial difference in the b* axes, indicating a shift from blue to yellow. The measurements also showed a positive difference in the a* axes (green to red), although to a smaller degree. Figure 4 shows the ΔE values for the treatment groups, in relation to control group 2. Treatment groups 1 (TPA6) and 2 (TPA7) had a ΔE value of 11.55 and 10.55 respectively.

**Figure 5:**
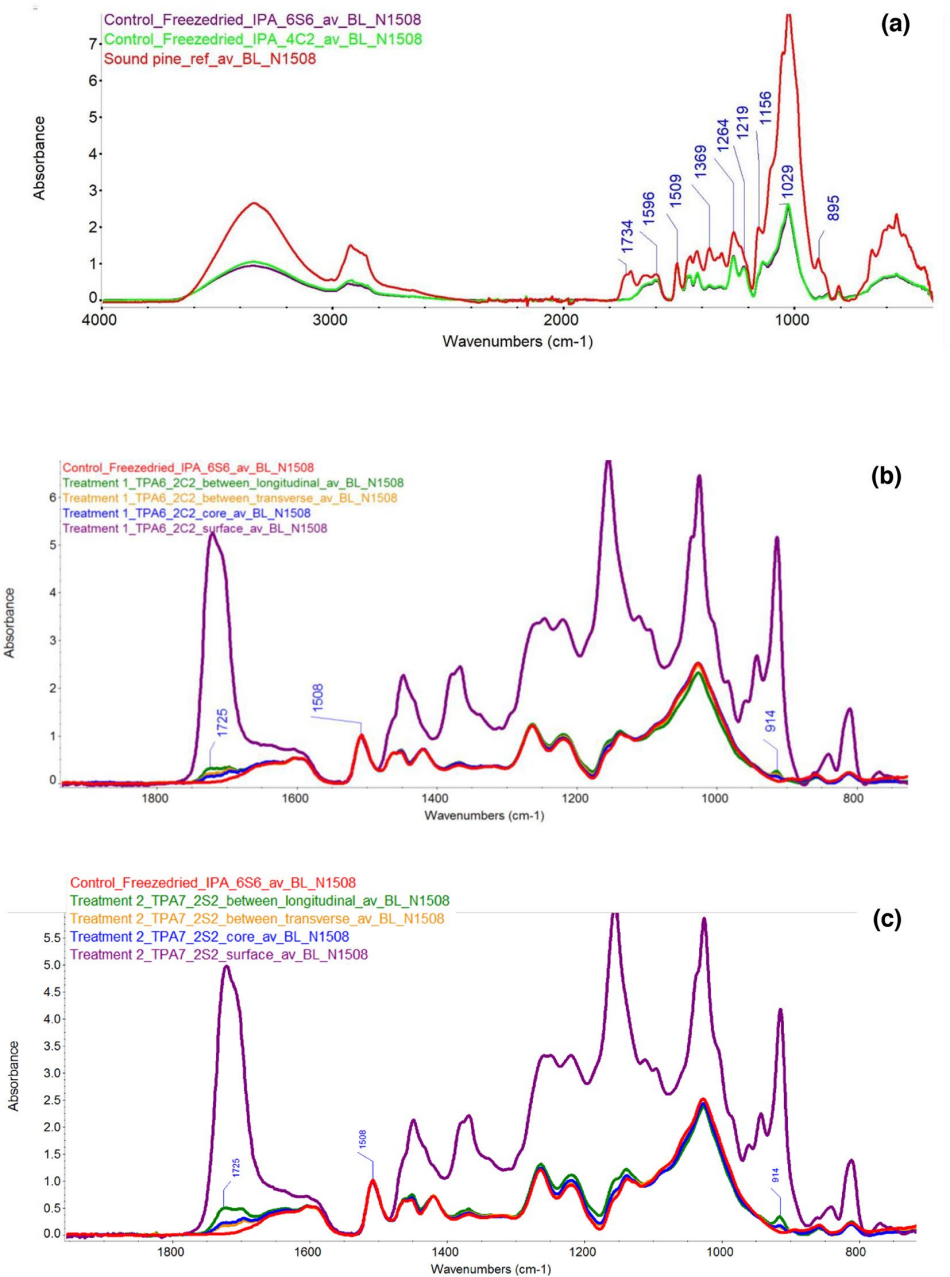
(**a**) IR spectra of sound pine (red) and archaeological pine (purple; isopropanol immersed surface control 6.S.6 and green; isopropanol immersed core control 4.C.2). The major peaks of sound wood are annotated. The spectra were normalised at 1508 cm^−1^. (**b**) IR spectra comparison (fingerprint region) between the isopropanol-immersed control (red; 6.S.6) and the samples treated with TPA6 (2.C.2) (**c**). As (**b**), but showing the TPA7-treated samples (2.S.2). The treated samples were measured at different depths: at the surface (purple), core (blue), between the surface and the core along the grain (green) and between the surface and the core across the grain (yellow). The signals depicting polymer penetration are annotated. Spectra were normalised at 1508 cm^−1^.

An ΔE above 3 can generally be seen by the human eye^21^. However, the colour change caused by these polymers may possibly not be detected if applied to the alum-treated Oseberg collection, since the artefacts are already dark^4^. As such, such changes would be considered acceptable. The final colour of the re-conserved collection should ideally not differ dramatically from that before re-conservation. Additionally, if different retreatment methods are applied to different parts of the collection (for example, aqueous treatments for those that can withstand water and non-aqueous methods for the remaining alum-treated objects), the resulting changes in colour should be similar. Thus, future work must include application of these polymers to test fragments of original Oseberg material, in order to evaluate colour change.

### ATR-FTIR analyses

The archaeological wood controls were first compared to sound wood, in order to better understand their state of degradation. Figure 5a shows such a comparison, with the use of a surface sample 6.S.6 (isopropanol-immersed control) and a core sample 4.C.2 as the archaeological wood. Normalisation was carried out at the lignin peak (1508 cm^−1^). One could see that the cellulose peaks (1369, 1156 and 895 cm^−1^)^22,23^ were muted in the archaeological wood, indicating its state of degradation and signals for the hemicellulose peaks at 1734 and 1239 cm^−1^ were not visible^22^. The lignin signals at 1596, 1264 and 1219 cm^−1^ were prominent in the archaeological wood, mainly due to their amplification as a result of the loss of cellulose and hemicellulose^22^. The band at 1029 cm^−1^ is due to a combination of several wood components (cellulose, hemicellulose and lignin)^23^ and as such, it shows the greatest absorption in both sound and archaeological pine.

Figure S5 shows a comparison of the IR absorbances of pure TPA6 and TPA7, normalised at 1725 cm^−1^ (C=O bond). The spectra of the two polymers were almost identical to one another. This was expected as they are structurally very similar. The major difference between the polymers was at 2856 cm^−1^, where TPA7 had a more pronounced peak. This could be attributed to a C–H stretching vibration, possibly due to the oleic acid component of the copolymer. The absorbances of the two pure polymers were then compared to those of a control specimen. The spectra showed that both polymers were easily distinguishable from the wood, although some signals overlapped, such as at the 3500–3000 cm^−1^ region (Fig. S6). The polymers had identifiable signals at 3000–2900 cm^−1^, as well as at 1716 and 1154 cm^−1^ (assigned as C=O and C–O respectively). Due to these differences, it was expected that the polymers would be easily distinguished from the wood in the treated samples.

The wood specimens treated with the polymers were analysed at different depth points. This was done to be able to get an indication of the level of distribution of the polymer through the entire specimen. Samples were taken from the surface, core, between the surface and the core along the grain and between the surface and the core across the grain. Their spectra were then compared to those of an untreated, isopropanol-immersed control (Fig. 5b,c). The spectra were normalised at the lignin peak at 1508 cm^−1^ as this was shared by all the samples. Figure 5b,c show that the specimen surface spectra were very different from all the other signals, clearly showing a high concentration of polymer. The rest of the treated wood specimens were more similar to the control, however they still showed a clear indication of polymer penetration. This was seen at 1725 and 914 cm^−1^ in particular. The spectra taken from the core of the treated specimen had the least absorbance.

The sample taken from in between the surface and core absorbed less than the surface but more than the core. The sample taken along the grain had more absorbance than the one across the grain, as can be seen at both 1725 and 914 cm^−1^. This was somehow expected since it would be easier for the polymer to penetrate along the grain of the wood rather than across it. The decreasing degree of polymer penetration (both TPA6 and TPA7) could therefore be described as follows: surface > between surface and core (along the grain) > between surface and core (across the grain) > core.

### SEM analyses

Cross-sections of latewood were primarily looked at, as opposed to earlywood. This is because generally, morphological changes appear more prominently in latewood due to its thicker cell walls. Sound pine was first compared to an archaeological wood control. The sound pine image showed that the cells clearly had a more robust shape, with regular and thick cell walls. In contrast, the archaeological wood had cells which appeared ‘feathery’ and fragile, with very thin cell walls appearing to be mostly dislodged (Fig. S7) This may be due to the loss of holocellulose (the term for combined cellulose and hemicellulose) previously depicted by the FTIR analyses.

**Figure 6:**
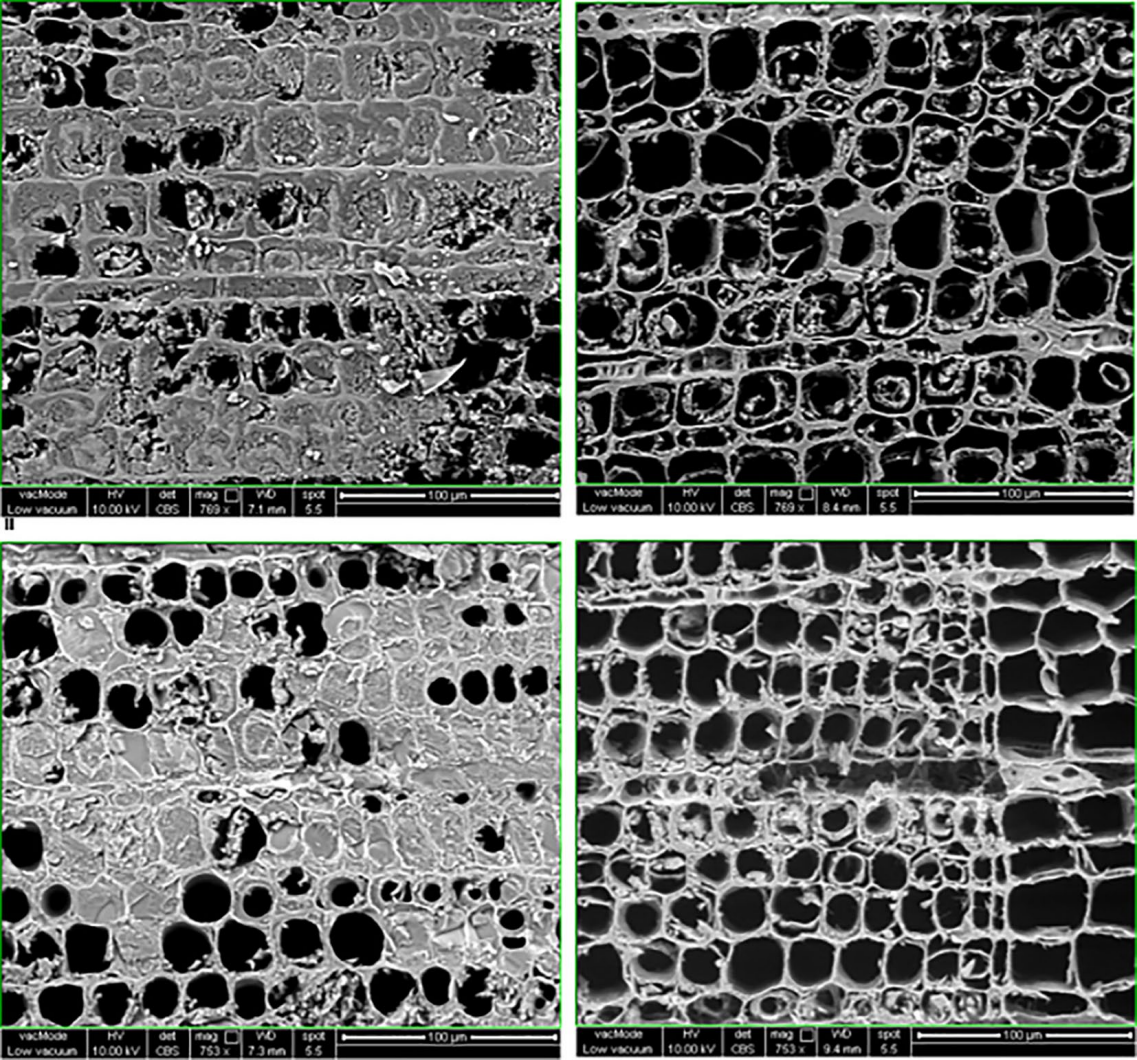
SEM images of samples treated with TPA6 (top), taken from the surface (top left) and core (top right). Bottom figures are the SEM images of samples treated with TPA7, taken from the surface (bottom left) and core (bottom right). The surface images show a high distribution of polymer, which filled many wood cells. The polymers were more difficult to observe in the core images, but the cells appear to be more structurally sound than the untreated sample, indicating polymer presence.

Samples from wood specimens treated with both TPA6 and TPA7 (Fig. 6) were taken. For each specimen, a sample was taken from the surface and the core. It should be noted that generally, it is difficult to identify organic polymers such as TPA6 and TPA7 from other organic materials such as wood with SEM. The specimens treated with both polymers showed similar results. The polymer was clearly visible in the surface samples, in some cases clogging the wood pores or lumina.

The polymer was more difficult to discern in the samples taken from the cores. It could be noted however that the treated cores appeared to be less airy than the control, with some cells having slightly thicker cell walls. They also seemed to be less deformed and appeared to be able to withstand the shaving that was carried out on the samples more than the control, possibly indicating an increased resilience. It was however difficult to say for certain whether this was due to the presence of the polymer.

In general, the SEM analyses appeared to correlate with the FTIR results. Both techniques showed that the highest concentration of polymer was on the surface of the wood specimens. FTIR indicated that the polymers managed to penetrate the cores of the specimens, albeit at a much lower concentration. This was difficult to observe with the SEM, but there were possible morphological changes that could perhaps indicate the presence of the polymers.

### Hardness test

**Figure 7:**
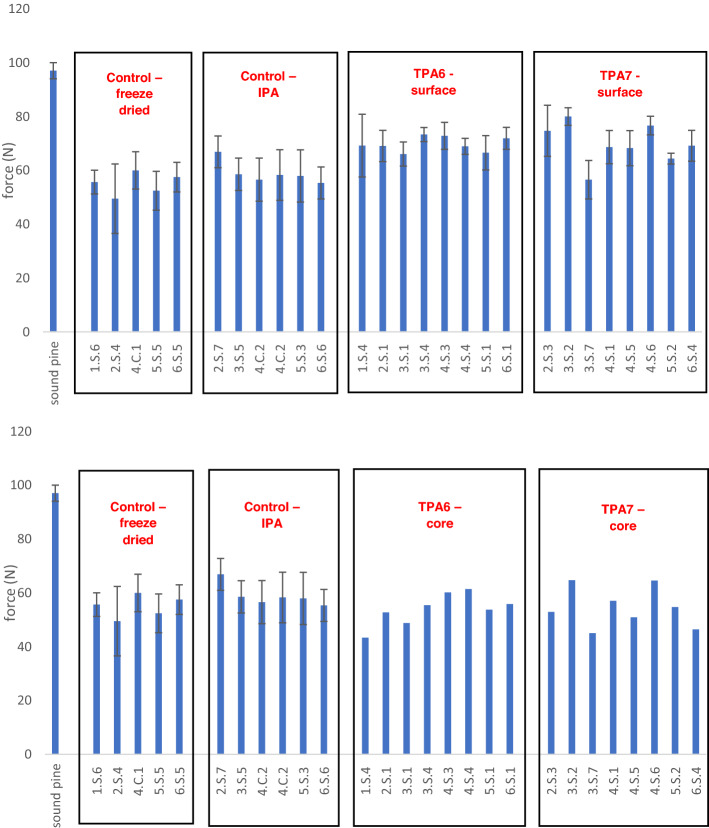
A comparison of the results from the hardness tests. The values for the treatment groups show the surface measurements (top) and the core measurements (bottom). The values for the treated cores do not have error bars since only two measurements were taken per sample.

The hardness tests were carried out in order to get an indication of the extent of consolidation that the polymers conferred. The fruit penetrometer measures the wood’s resistance to indentation. Specimens from control group 1 (freeze-dried only) and control group 2 (freeze-dried and immersed in isopropanol) were measured five times on the tangential surface. Specimens from both polymer treatment groups were measured five times on the tangential surface. In addition, each of the treated specimens was split in half and two measurements were taken from the core (one measurement per half piece, totalling two measurements of each core). The multiple measurements taken from every specimen were then averaged (Fig. 7).

From the averaged values, one could see that there seemed to be an increase in surface hardness compared to the controls. Conversely, the core hardness did not appear to change in the treated groups. Statistical analysis with the one-way ANOVA was carried out on the surface measurements of the treated groups in order to determine whether the observed change in hardness was significant. This was done using the individual measurements instead of the averaged values.

The control group used for the statistical analysis was made up of both the freeze-dried and isopropanol-treated controls. This was justified since the difference in means between the two groups was not significant (Fig. S8). There were 40 values in the control group, 40 values in the TPA6 treatment group and 40 values in the TPA7 treatment group. There were two outliers in the TPA6 treatment group, as assessed by a boxplot. It was decided to not remove these outliers from the dataset.

The data was normally distributed except for the TPA7 group, as assessed by the Shapiro–Wilk test (*p* > 0.05). However, the one-way ANOVA is known to be robust to deviations from normality, especially if the sample sizes are equal (as was the case here)^24^. The Levene’s Test indicated that homogeneity of variances was violated (*p* = 0.025), therefore the Welch’s one-way ANOVA was used for this analysis. The surface hardness score was found to be statistically significantly different for the two groups treated with the different polymers (Welch’s *F*(2, 1641.693) = 33.258, *p* < 0.001) (Fig. S9).

The surface hardness increased from the control (58.7 ± 6.8) to the TPA6 treatment group (69.8 ± 6.4) and the TPA7 treatment group (69.8 ± 8.9). Games-Howell post hoc analysis revealed that the increase from the control to the TPA6 treatment group (11.0875, 95% CI (7.561 to 14.614)) was statistically significant (*p* < 0.001), as well as the increase in surface hardness for the TPA7 treatment group (11.1050, 95% CI (6.855 to 15.355), *p* < 0.001). The difference between the groups treated with the different polymers was not statistically significant (0.0175, 95% CI (0.0 to 4.174), *p* = 1.00).

This analysis indicated that the polymers imparted a clear increase in surface hardness, possibly making the wood more resistant to stresses caused by handling or the environment. Both TPA6 and TPA7 seemed to increase the hardness to the same extent, as there was no statistical difference between them. Conversely, the cores of the treated samples did not appear to increase in hardness but this was expected, as there was only a small amount of polymer present compared to the surfaces.

### TPA6 or TPA7?

This preliminary study aimed to determine the extent of penetration of two terpene-derived polymers, TPA6 and TPA7, in highly degraded archaeological pine. This was carried out by monitoring weight and dimensional change, alongside the use of ATR-FTIR supplemented with SEM. Other tests were carried out to observe the effects that the polymers had on the wood, such as colour change measurements and hardness tests.

TPA6 and TPA7 showed very similar results and they seemed to affect the wood in mostly the same way. Generally, all the treated specimens increased in weight, indicating polymer uptake. Slight dimensional changes were also recorded for all specimens. The colour of the polymer-treated wood was slightly darker, with a yellowish tinge. FTIR showed that the polymers successfully penetrated the wood, with the highest concentration on the surface. This correlated well with the SEM analyses, which showed an abundance of polymer on the surface of the wood specimens. Both polymers also penetrated the core of the wood specimens, although to a lesser extent, making them difficult to pinpoint in the SEM images. Hardness tests proved that the surface of the treated specimens was significantly harder than the controls and therefore more resistant to indentation. Overall, the differences between the two polymer treatment groups were not significant, as was observed for all the tests that were carried out. Because of this, TPA6 should be favoured over TPA7, due to its higher *T*_g_ and since its synthesis possesses fewer steps and is therefore less time-consuming.

## Concluding remarks

Compared to other experimental consolidants currently being investigated, TPA6 and TPA7 appear to be very promising. Colour change proved to be not as drastic as reported for other consolidants like soda lignin^4^. Other investigated materials such as isoeugenol^8,9^ and aminocellulose^11^ have yet to be proven to have a consolidative effect. Conversely, the hardness tests described in this study have shown that TPA6 and TPA7 improve the wood’s resistance to indentation. One of the possible limitations of these terpene-derived polymers is the fact that they have been shown to plug the surface wood lumina, as shown by the SEM results. This could possibly hinder any future re-treatment attempts of the wood. Moreover, these polymers did not appear to increase the hardness of the wood cores. This was expected since the IR results did not indicate a large degree of polymer penetration into the cores.

Due to the promising results, the general scope of this study can be expanded in future work. A new, more accurate method of measuring dimensional change should be developed. A possible idea is to make markings on the wood with a pen before treatment, and then measure the dimensional changes with a technical ruler as opposed to callipers. Higher concentrations of polymer can be used to determine whether this would make a difference in the extent of penetration to the wood core. The immersion time can also be prolonged as this may also potentially increase the amount of polymer that manages to penetrate to the core. Other different methods of polymer administration can also be explored, such as injection. Additionally, these tests should be run using higher sample numbers of archaeological wood in order to increase the robustness of these studies. Long-term studies should also be carried out to determine the degree of stability of the polymers over time and to see whether they interact with alum and whether they are stable in the presence of the remaining acidity in wood (target pH 4–5) after deacidification. Moreover, the effects of relative humidity both on these polymers in their pure form and after wood and alum penetration, should be investigated. This can be done by monitoring weight changes at different RH levels. If the results from these tests prove to be promising then the polymers may pass into the next phase of investigation, where they will be tested on alum-treated Oseberg samples, using the same methods applied here. Finally, if the polymers show promising results, work must then focus on optimising the scaling-up process to obtain larger quantities of material. Here we must also focus on ensuring the polymer batches are consistent and predictably synthesised.

## Supplementary Information

Supplementary Figures.

## Data Availability

Raw data is available in Supplementary Information and on request from the Corresponding Authors.
