# Supplementary material for: Evaluation of two terpene-derived polymers as consolidants for archaeological wood
Source: Sci Rep. 2023 Mar 4;13:3664. doi: 10.1038/s41598-023-29785-5 (PMC9985608; doi:10.1038/s41598-023-29785-5)
Supplement: Supplementary file 1 — Supplementary Figures. [file 41598_2023_29785_MOESM1_ESM.docx]

**Evaluation of two terpene-derived polymers as consolidants for archaeological wood**

Michelle Cutajar^1,2*^, Susan Braovac^3^, Robert A. Stockman^2^, Steven M. Howdle^2^ and Stephen E. Harding^1,3*^

1. *National Centre for Macromolecular Hydrodynamics (NCMH), University of Nottingham, School of Biosciences, Sutton Bonington, LE12 5RD, U**K*
2. *School of Chemistry, University of Nottingham, University Park, Nottingham, NG7 2RD UK*
3. *Museum of Cultural History, University of Oslo, Kabelgata 34, 0580 Oslo, Norway*

*Corresponding authors: [michelle.cutajar@nottingham.ac.uk](mailto:michelle.cutajar@nottingham.ac.uk) and [steve.harding@nottingham.ac.uk](mailto:steve.harding@nottingham.ac.uk)

**Supplementary Material**


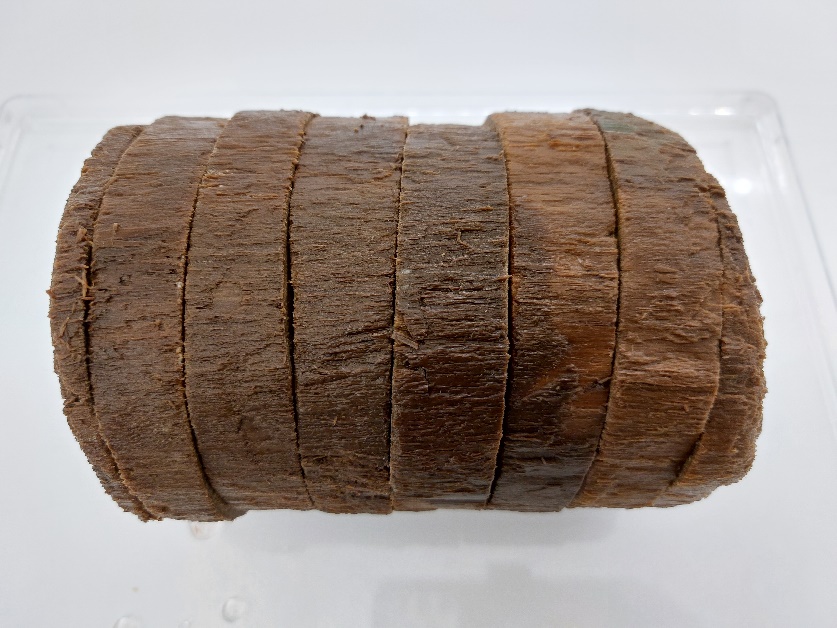


**1**

**2**

**3**

**4**

**5**

**6**


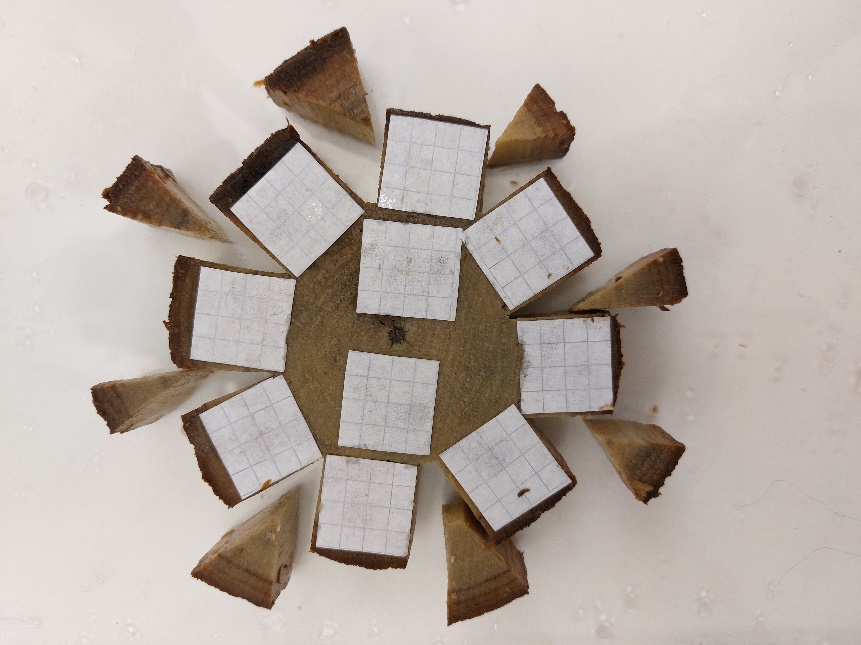


**S**

**S**

**S**

**S**

**S**

**S**

**S**

**S**

**C**

**C**

**Figure S1.** The original wood log which was used to cut the slices, numbered 1 to 6 (left) and the process of sawing off the specimens from one of said slices (right). The graph paper shows squares which are 2 x 2 cm^2^. Each specimen is labelled S or C, indicating if it was cut from the surface (S) or the core (C) of the slice.


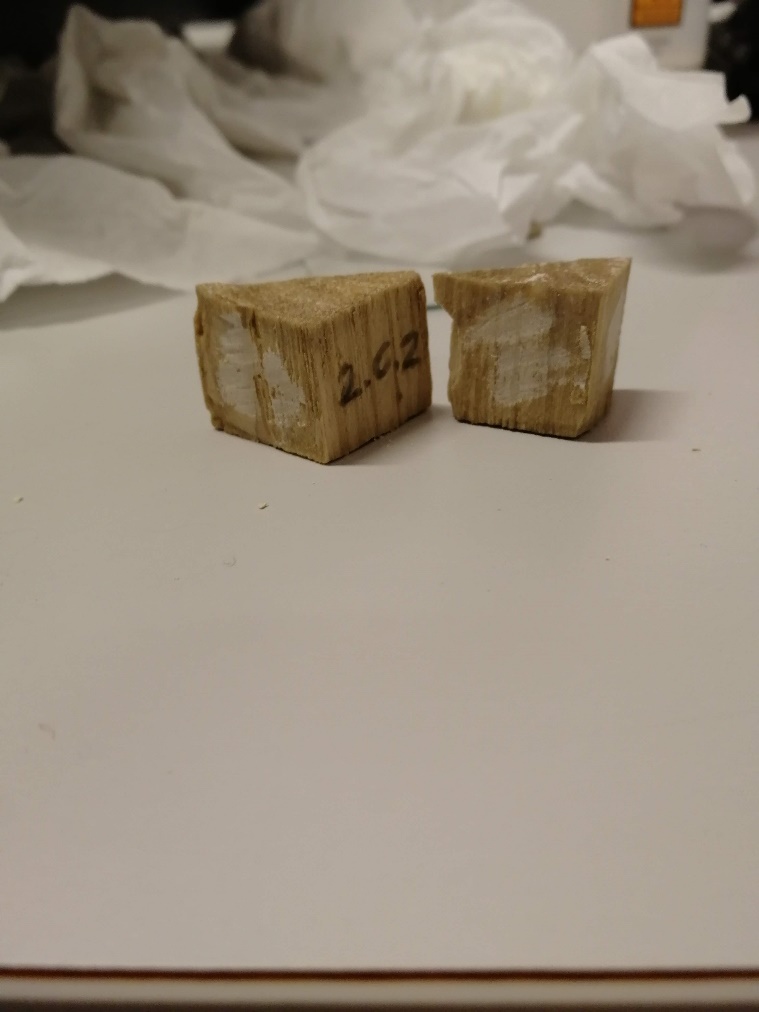


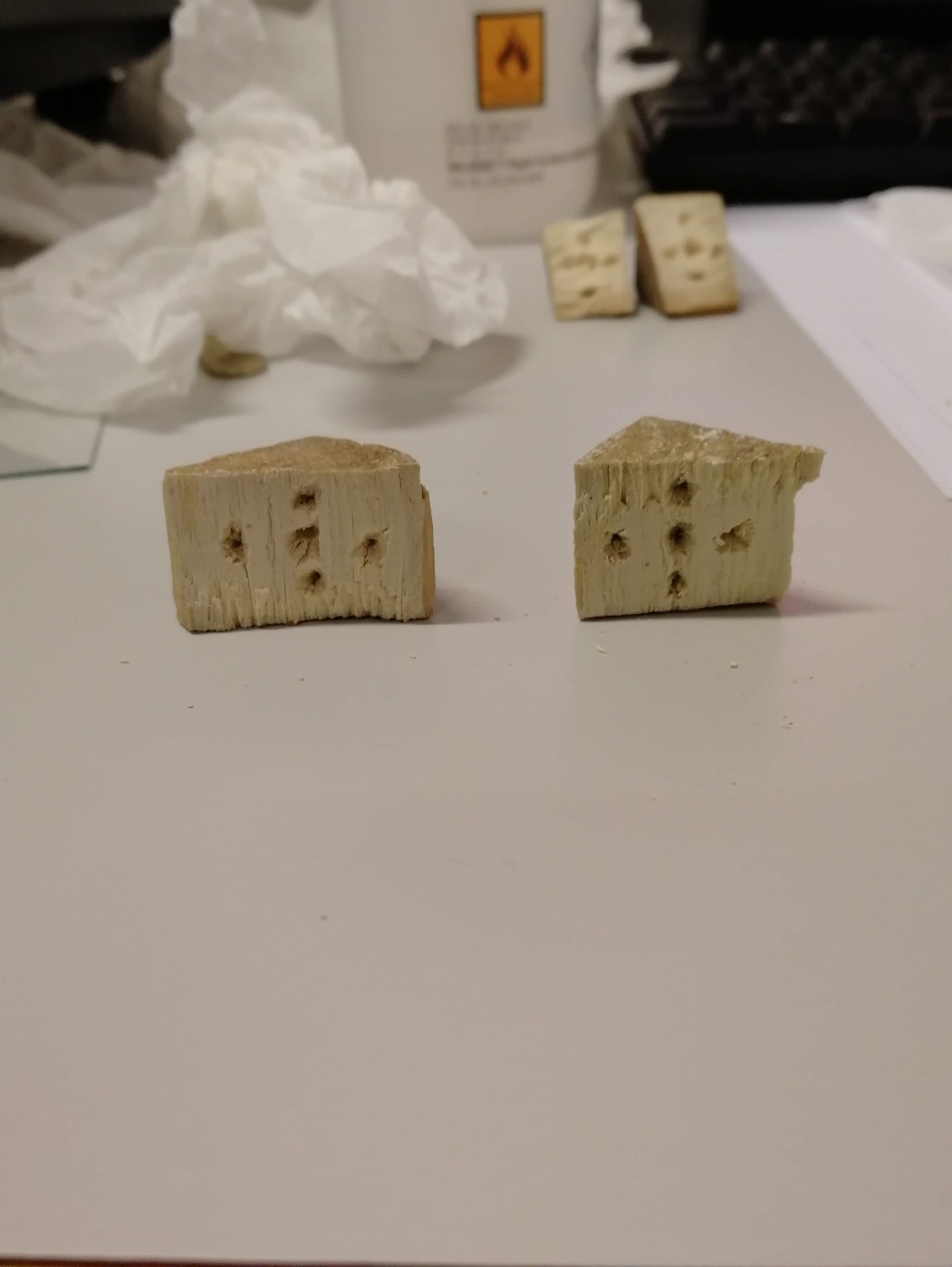


**Between surface and core – along the grain**

**Between surface and core – across the grain**

**Core**

**Surface**

**Figure S2.** The different sampling locations for IR. The image on the left is showing how each wood cube was split in the middle, whilst the image is indicating where the samples were taken.


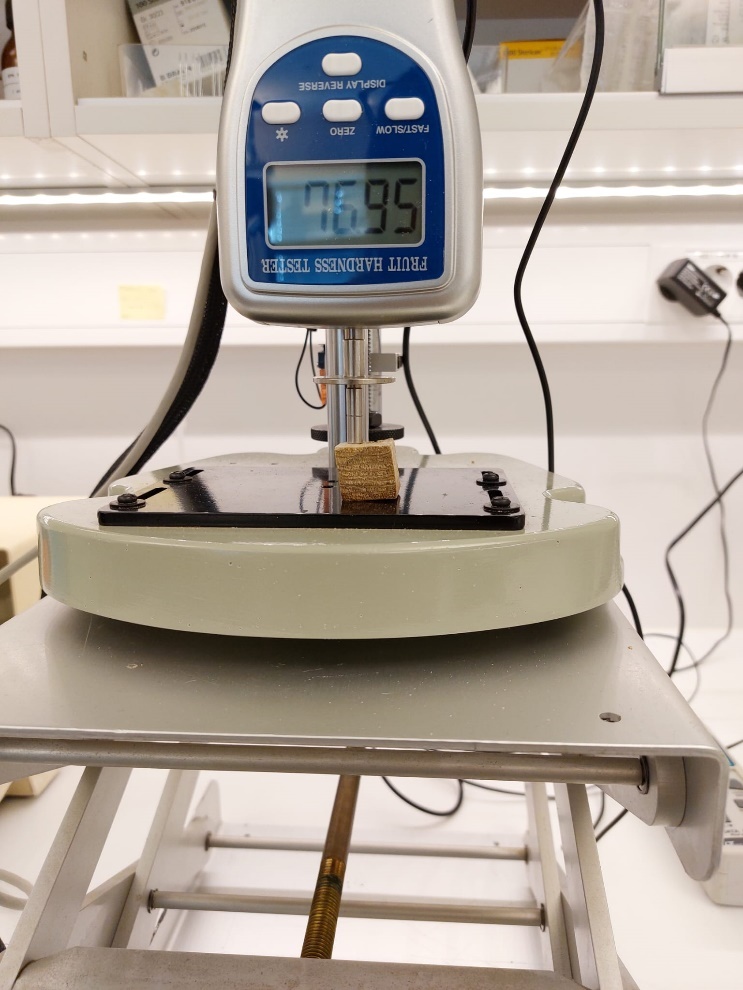

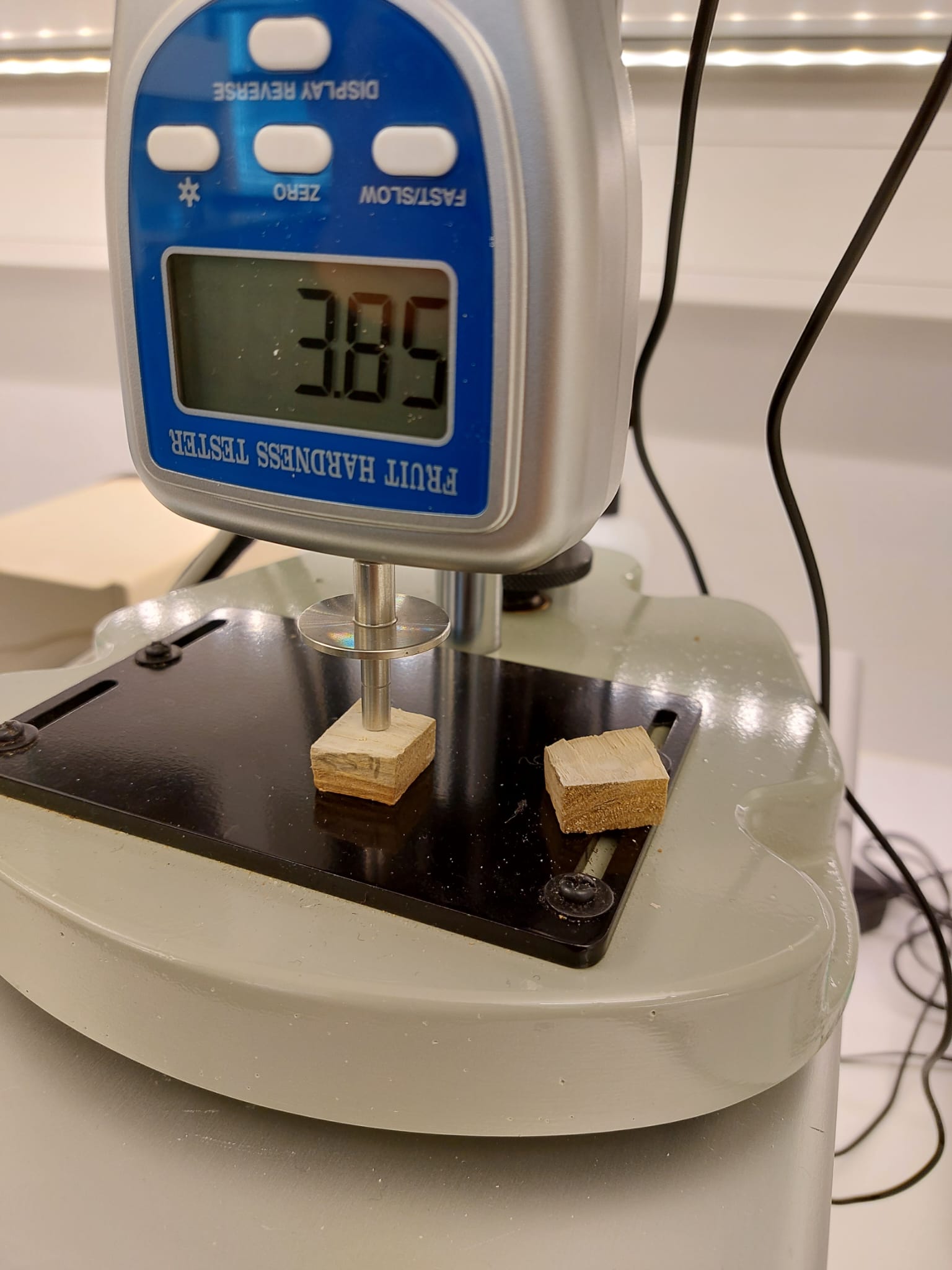


**Figure S3.** The fruit penetrometer used for the hardness test. Photos show the measurement of surface (left) and core hardness (right) of treated specimens.

**Figure S4**. A comparison of the % dimensional changes of control groups 1 and 2 and treatment groups 1 and 2. The treatment groups show a % weight gain when compared to the controls. Conversely, the % dimensional changes do not appear to have varied greatly after treatment.

**
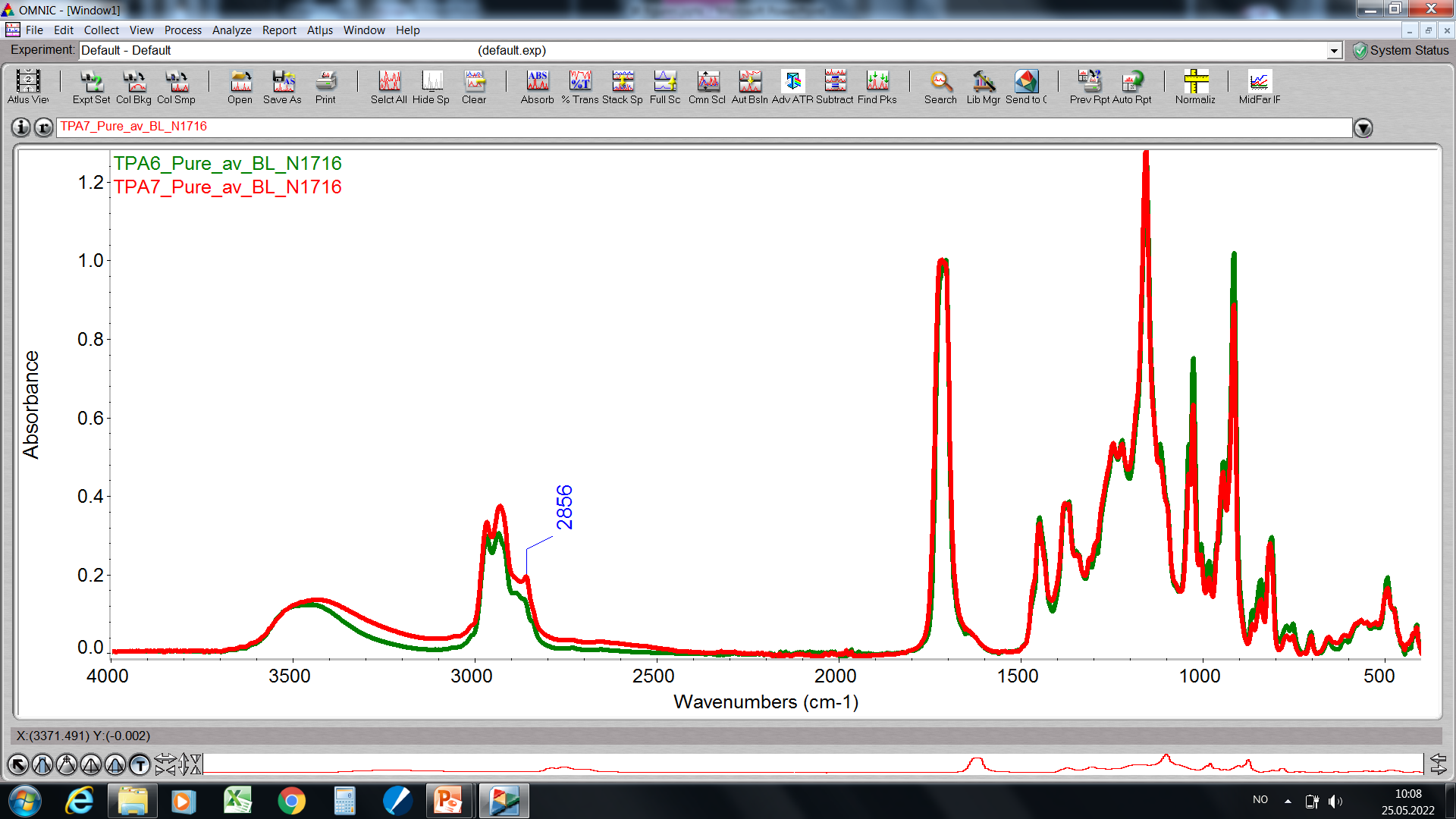
**

**Figure S5**. IR spectra of TPA6 (green) vs TPA7 (red). The spectra were normalised at 1725 cm^-1^.


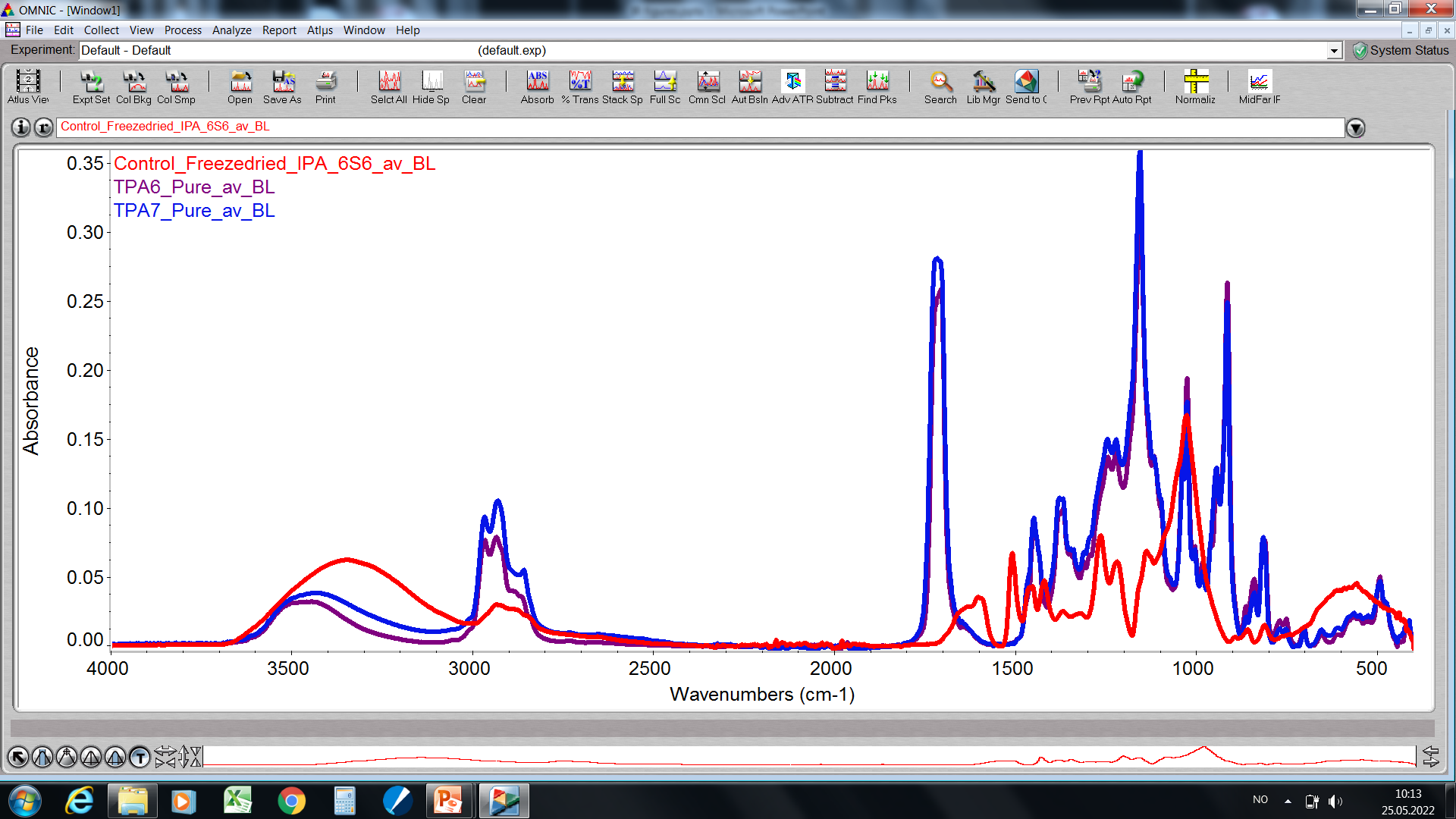


**Figure S6**. The spectra of the TPA6 (purple) and TPA7 (blue) vs archaeological wood (red; isopropanol immersed control 6.S.6). The spectra were not normalised.


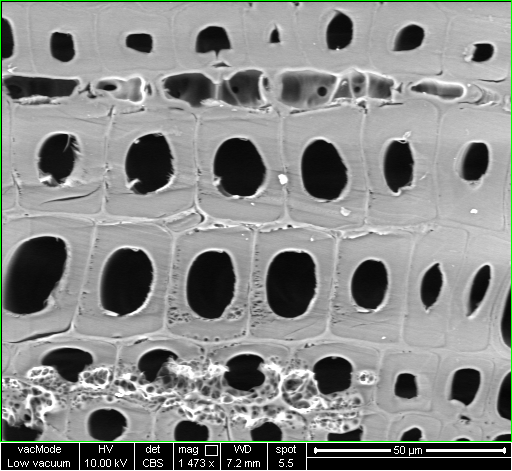
**
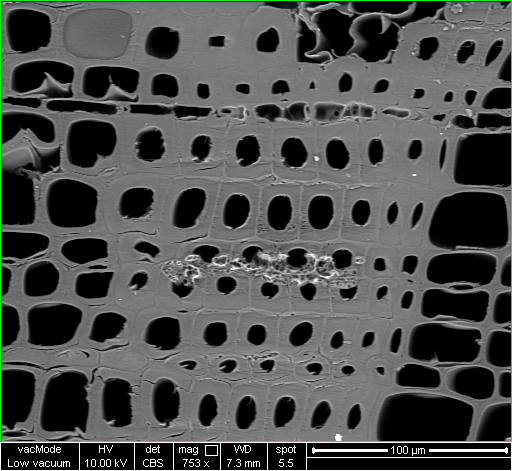
**


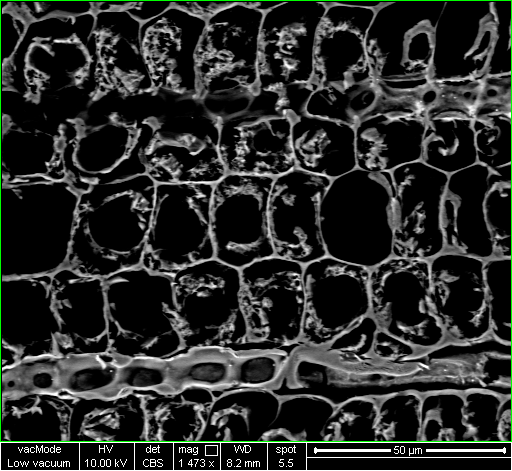
**
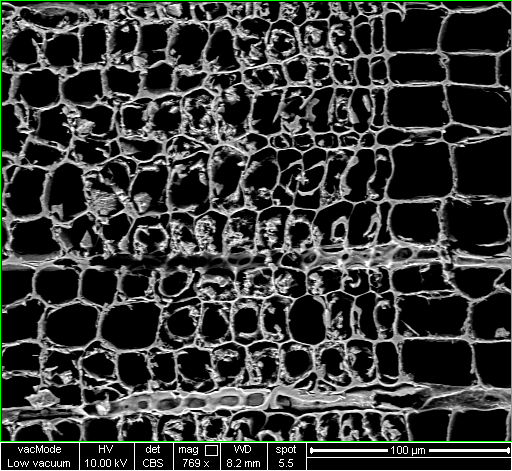
**

**Figure S7**. SEM images of sound pine (top) versus archaeological, isopropanol-immersed wood (bottom). The images on the right were taken with a higher magnification. The sound wood has regularly spaced cells with thick cell walls while the archaeological wood has lost most of its structure.


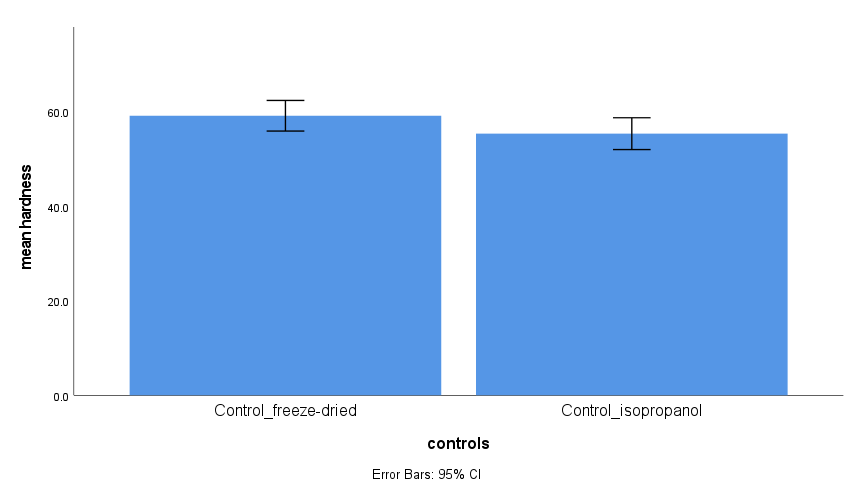


**Figure S8**. Results for the independent t-test run on the two control groups. The isopropanol-treated controls were harder (59.0 ± 7.8) than the freeze-dried only controls (55.3 ± 8.1), with a difference of 3.78 which was not statistically significant (95% CI, 0.00 to 8.32), *t*(48) = 1.673, *p* = 0.101.


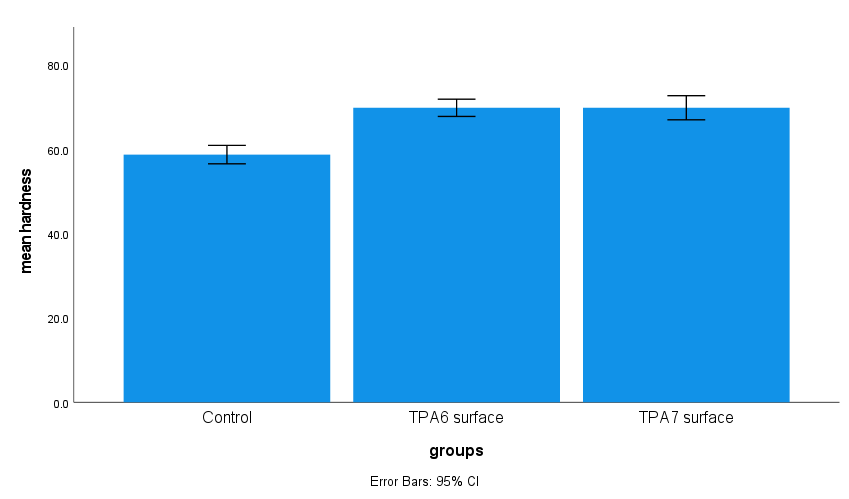


**Figure S9**. Results for the one-way ANOVA run on the treatment groups surface measurements. The hardness score was statistically significantly different for the sample groups, Welch's *F*(2, 1641.693) = 33.258, *p* < 0.001.
